# Profile and mortality outcome of patients admitted with cryptococcal meningitis to an urban district hospital in KwaZulu-Natal, South Africa

**DOI:** 10.7448/IAS.17.4.19623

**Published:** 2014-11-02

**Authors:** Benjamin Adeyemi, Andrew Ross

**Affiliations:** 1 Family Medicine, Pietermaritzburg Hospitals Complex and University of KwaZulu-Natal, KwaZulu-Natal, South Africa; 2 Family Medicine, University of KwaZulu-Natal, KwaZulu-Natal, South Africa

## Abstract

**Introduction:**

Cryptococcal meningitis (CCM) is one of the leading causes of early mortality among HIV-infected patients. This study was a part of clinical audit [1] aimed at improving care for patients with CCM at an urban district hospital in South Africa.

**Methods:**

Clinical records of all patients (age>13 years) admitted to the hospital with a diagnosis of CCM (based on a positive India ink, positive cryptococcal latex agglutination test (CLAT) or a positive culture of *Cryptococcus neoformans*) between June 2011 and December 2012 were retrospectively reviewed. Descriptive statistics and Chi-square analysis were generated with Epi Info 7.1.2.0. 95% confidence intervals were reported where appropriate.

**Results:**

Of the 127 patients admitted with CCM, only 97 (76.4%) knew their HIV status. Only 44.8% (43/96) of those who knew they were HIV positive were on antiretroviral therapy (ART). Seventeen out of 25 patients (68%) previously treated for CCM had defaulted fluconazole and only 60% (15/25) were on ART. Acute mortality (death within 14 days of CCM diagnosis) was 55.9% (71/127). The median time to death from diagnosis was four days (IQR 2–9). The association between CSF WBC count<20cells/mL and increased risk of death within 14 days was statistically significant (OR 2.2; 95% CI 1.1–4.6, p=0.03). Patients with heavy cryptococcal burden (reported as numerous yeasts seen on microscopy) at diagnosis were three times more likely to die within 14 days of diagnosis of CCM (OR 3.2; 95% CI 0.9–10.7, p=0.06). Even though a CD4 count<100cells/mm^3^ was associated with a 1.6 times increased acute mortality risk, the association was not statistically significant (OR 1.6; 95% CI 0.6–4.6, p=0.3). The role of elevated CSF opening pressure at diagnosis was not assessed because only two (1.6%) patients had their baseline opening pressure measured.

**Conclusions:**

Acute CCM-related mortality remains high. The number of patients who do not know their HIV status, the number of HIV positive patients not on ART, the high level of non-adherence to fluconazole and the proportion of patients not on ART after at least one previous CCM episode all point to the need of developing comprehensive strategies aimed at encouraging HIV testing and improving patient's retention in HIV care and support.
